# Successful Biliary Re‐Cannulation and Neo‐Anastomosis Creation in Complete Bile Duct Occlusion or Disruption Using a Combination of Interventional Radiology and Endoscopic Techniques: A Case Series

**DOI:** 10.1002/jgh3.70278

**Published:** 2026-02-24

**Authors:** Joshua G. Fricker, Tarik Babar, Husameddin El Khudari, Ali Ahmed, Dalton Norwood, Sergio A. Sánchez‐Luna, Eric Bready, Ramzi Mulki, Kondal Kyanam, Shajan Peter, Andrew Gunn

**Affiliations:** ^1^ Department of Internal Medicine University of Alabama at Birmingham Birmingham Alabama USA; ^2^ Department of Interventional Radiology University of Alabama at Birmingham Birmingham Alabama USA; ^3^ Department of Gastroenterology and Hepatology University of Alabama at Birmingham Birmingham Alabama USA; ^4^ Department of Preventive Medicine University of Alabama at Birmingham Birmingham Alabama USA

## Abstract

**Aims:**

Biliary strictures are a known complication of hepatobiliary and pancreatic surgeries. This retrospective descriptive series highlights six consecutive patients with complete bile duct occlusion or disruption who required a multidisciplinary approach with a combination of interventional radiology (IR) and endoscopic techniques to either re‐cannulate or create a neo‐anastomosis in the biliary system.

**Methods and Results:**

The biliary system was either re‐cannulated or a neo‐anastomosis was created using a small gauge needle (*N* = 3), a rendezvous procedure (*N* = 1), a radiofrequency ablation wire (*N* = 1), and the back end of a wire (*N* = 1). There was one major adverse event (bacteremia requiring antibiotic therapy). Follow‐up protocol included scheduled biliary catheter checks every 10–12 weeks to assess biliary duct patency or tract maturation. At each interval, cholangioscopy and cholangioplasty were performed as indicated. Once the tract demonstrated maturity and liver function tests normalized, the catheter was converted to an external‐only drain. Patients who successfully tolerated a capping trial of the external biliary catheter subsequently underwent catheter removal. Patients who remained asymptomatic with normal liver function tests following catheter removal were considered to have achieved catheter‐free status.

**Conclusion:**

Four of the six patients are biliary catheter free, while two of the patients are progressing toward being catheter free. Additional data from larger series and planned prospective registries should help delineate standards for outcomes and patient management.

## 1 Introduction

Biliary strictures are a known complication of hepatobiliary and pancreatic surgeries and can be seen in up to 5% of patients after a Whipple procedure [1, 2]. Total biliary occlusion or disruption is a challenging complication that requires a multidisciplinary approach. Interventional radiology (IR) plays an important role in filling the care gap in these difficult clinical situations. This brief report describes six patients with complete biliary occlusion or ductal disruption that were treated with either sharp re‐cannulation or image‐guided neo‐anastomosis creation. Patients included in this series had complete bile duct occlusion or disruption with no plans for surgery. We describe the various interventional radiology (IR) and advanced endoscopic techniques employed for successful biliary recanalization or neo‐anastomosis creation. We also report on clinical outcomes, procedure‐related complications, and patient follow‐up. Technical success was defined as either successful recanalization of the occluded or disrupted bile duct or creation of a neo‐anastomosis, followed by radiologic confirmation of effective biliary drainage into the small bowel through a placed biliary catheter. Clinical success was defined as normalization of liver function tests in patients who initially presented with clinical symptoms or abnormal liver function (Table 1).

### 1.1 Cases

#### 1.1.1 Patient 1

55‐year‐old male with a history of metastatic neuroendocrine tumor (NET) to the liver who was treated by right hepatectomy and wedge resection on the left. During post‐operative follow‐up, he was noted to have elevated total bilirubin levels, which prompted cross‐sectional imaging that demonstrated mild ductal dilatation in segments 2 and 3. IR was consulted for percutaneous transhepatic cholangiography (PTC) with drain placement, during which the common bile duct (CBD) was not seen, and an external biliary drain was placed. MRCP showed disconnection between the ducts of segments 2 and 3 and the segment 4 duct, which connected to the CBD. Subsequently, PTC was performed to place an internal/external biliary drain through segment 4. During a separate procedure, fluoroscopic guidance was used to pass a 21G needle from the segment 4 access across the hepatic parenchyma and into the segment 2/3 ductal system, where the 0.018 wire was snared for through‐and‐through access. IR dilated the intra‐hepatic tract with a 7 mm balloon to place a 14F internal/external biliary drain that connected the entire biliary system. Over the course of 9 months, he underwent serial cholangioplasty and drain exchange every 10–12 weeks. Then, to test the tract, we exchanged the drain for a 4F Kumpe catheter. This catheter was left in place for 2 weeks and, after assuring normal liver function tests, was removed in clinic. The patient is noted to be doing well approximately 22 months after the initial procedure.

#### 1.1.2 Patient 2

50 year‐old female with metastatic anal squamous cell carcinoma to the liver who underwent left hepatectomy. During post‐operative follow‐up, her total bilirubin increased from pre‐procedure levels of 1.7 to ~9 mg/dL. This prompted an MRCP that showed right biliary ductal dilatation without dilatation of the CBD. An endoscopic retrograde cholangiopancreatography (ERCP) was performed, but the CBD could not be cannulated due to prior sleeve gastrectomy. IR was consulted for PTC, during which the CBD was not identified and an external biliary drain was placed. The patient was then set up for a rendezvous procedure with advanced endoscopy. The endoscopy team was able to cannulate the CBD and placed a snare for IR to use as a target. IR attempted to cross a transhepatic tract from the right biliary tree into the CBD with a 21G needle, but the angulation made this difficult. One of the fluoroscopically‐guided punctures took a transhepatic route to enter the duodenum directly. This was confirmed by both contrast injection and endoscopy. After dilatation of the tract, a 14F internal/external biliary drain was placed to help the tract mature. Over 5 months, she underwent serial cholangiography and drain exchange every 10–12 weeks to assess the maturity of the tract. Once cholangiography showed no spilling of contrast into the peritoneum, the drain was exchanged for a 4F Kumpe catheter to test the anastomosis. The catheter was removed in clinic approximately 2 weeks later after confirming that liver function tests were normal. The patient lived three more months catheter‐free before dying due to progression of her metastatic disease.

#### 1.1.3 Patient 3

59‐year‐old female with a history of pancreatic adenocarcinoma status post metallic stent placement by endoscopy who presented with elevated liver function tests. Cross‐sectional imaging shows bilateral biliary ductal dilatation and IR was consulted for PTC with drainage. Cholangiography showed occlusion of the metallic stent. This could not be crossed using traditional wire, catheter, and sheath maneuvers. Percutaneous cholangioscopy was unsuccessful in crossing the occlusion. Subsequently, the inferior aspect of the stent was targeted percutaneously with a 22G needle and wire was navigated cranially across the occlusion. This wire was then snared via the PTC access and exchanged for an Amplatz wire. From the PTC access, a 12F sheath was then advanced from cranial to caudal across the occlusion, after which a Glidewire easily went into the small bowel. After exchanging the Glidewire for an Amplatz wire, a 14F internal/external biliary drain was placed. The patient came back to IR twice over the following 2 months for cholangioplasty and debris removal using the percutaneous cholangioscope. The tube was removed in clinic after normalization of her liver function tests. The patient has been catheter‐free for approximately 5 months and repeat imaging demonstrates pneumobilia, consistent with stent patency.

#### 1.1.4 Patient 4

79 year‐old female with a history of recurrent choledocholithiasis necessitating the creation of a hepatico‐jejunostomy (H‐J) in 2014 who presented with lethargy and unintentional weight loss. Evaluation for these complaints revealed elevated liver function tests and bilateral biliary ductal dilatation, consistent with a benign stricture at the H‐J anastomosis. Endoscopy was unable to cannulate the CBD, so the patient was referred to IR for PTC with biliary drainage. Initially, a left PTC was placed, but the obstruction could not be crossed using traditional methods. The patient returned for a rendezvous procedure with endoscopy so IR could use their scope as a fluoroscopic target, which was unsuccessful. Subsequently, the endoscope was placed by advanced endoscopy within the small bowel, close to the hepaticojejunostomy and was used as a radiographic target for the radiofrequency (RF) wire. The RF wire passed from the CBD into the bowel, which was confirmed by both contrast injection and endoscope visualization. The wire was exchanged for an Amplatz wire via a Neff set, and a 14F internal/external biliary catheter was placed. Over the course of 4 months, the patient underwent cholangiography and cholangioplasty to assist with the maturation of the tract. At final cholangiography, there was patency of the tract and no evidence of bile leak. The patient was seen in clinic where liver function tests were normal, so the catheter was removed. She has now been catheter‐free for approximately 4 months while liver function tests continue to be stable.

#### 1.1.5 Patient 5

72 year‐old female with a history of distal gastrectomy and Roux‐en‐Y gastric bypass in early 2022, who presented with elevated liver function tests approximately 8 months after her surgery. Cross‐sectional imaging showed bilateral biliary ductal dilation. Due to her altered anatomy, IR was consulted for PTC with drainage. Fluoroscopic‐guided access to a right biliary duct was obtained with cholangiography showing obstruction at the ampulla. First, we attempted to cross the stricture using traditional wire, catheter, and sheath techniques, but this was unsuccessful. Eventually, we took the back end of a Glidewire and used it to puncture across the occlusion. A catheter was placed over the wire, and contrast injection showed entry into the small bowel. After dilatation of the tract, a 14F internal/external biliary catheter was placed over an Amplatz wire. The patient was placed into our institutional benign biliary stricture protocol, which consists of biliary intubation for 3–6 months with intermittent cholangioplasty and tube exchanges. It has been approximately 6 months since the initial procedure, and this patient still has her internal/external biliary drain in place.

#### 1.1.6 Patient 6

69‐year‐old male with a history of metastatic colon cancer to the right lobe of the liver status post multiple‐segment hepatectomy, who presented with elevated liver function tests 4 months post‐operatively. Cross‐sectional imaging demonstrated a large biloma and biliary duct dilation. A 12F biliary stent was placed into the CBD by the gastrointestinal (GI) service. Additionally, a drain was placed into the biloma by IR. Subsequent imaging showed a decreasing size of the fluid collection within the biloma with persistent biliary duct dilatation. PTC was then performed showing an occluded left bile duct. Multiple unsuccessful attempts were made to cross the occlusion. An external left biliary drain was left in place. One week later, cholangioscopy performed by GI showed a transected common hepatic duct. An additional external right biliary drain was placed. A rendezvous procedure was then performed. Two Glidewires were advanced from the right and left hepatic duct into the biloma cavity and were snared with the help of cholangioscopy. Custom side holes were created on a regular 10F ReSolve APD drain to allow for drainage of the intrahepatic biliary system without leakage into the biloma. Both drains were advanced into the small bowel. Contrast injection through the drains showed appropriate opacification of the small bowel, CBD, common hepatic duct, and intrahepatic ducts without opacification of the biloma. The patient is doing well 1 month post‐procedurally with the catheters still in place.

**TABLE 1:** Case characteristics.

	Patient #1	Patient #2	Patient #3	Patient #4	Patient #5	Patient #6
Age	55	50	59	79	72	69
Sex	M	F	F	F	F	M
Benign/Malignant	Benign	Benign	Malignant	Benign	Benign	Benign
Intervention technique	21G needle	21G needle	22G needle	RF wire	Back end of wire	Rendezvous procedure
Re‐cannulation or neo‐anastomosis	Neo‐anastomosis	Neo‐anastomosis	Re‐cannulation	Re‐cannulation	Re‐cannulation	Neo‐anastomosis
Anesthesia	MAC	General	General	General	MAC	MAC
Technical success	Yes	Yes	Yes	Yes	Yes	Yes
Major AE	No	No	No	No	No	Bacteremia
Clinical success	Yes	Yes	Yes	Yes	Yes	Yes
Drain placement duration	9 months	5 months	3 months	4 months	Currently undergoing benign biliary protocol 6 months post‐procedurally	Currently still has drains 1 month post‐procedurally

Abbreviations: AE, Adverse event; MAC, Monitored Anesthesia Care.

## 2 Discussion

In this brief report of six patients with either total biliary occlusion or ductal disruption, a combination of advanced IR and endoscopy techniques was used. Four out of the six patients are catheter free, with the other two patients progressing toward being catheter free. The only major adverse event was in Patient 6, who had post‐procedural bacteremia treated with antibiotics. This report adds to the small, but growing, body of literature surrounding advanced IR techniques for biliary injuries and biliary pathology [3, 4].

## Consent

Informed consent was obtained from the patient for the publication of their information and/or imaging.

## Conflicts of Interest

The authors declare no conflicts of interest.

## Supporting information


**Figure S1:** (A) Cholangiogram through a right sided external biliary drain in Patient 3 showing intrahepatic bile duct dilatation and complete occlusion of bile ducts (arrow). (B) Percutaneous access of occluded stent using 21G needle and 0.018 wire advanced through needle toward the biliary system (arrow). (C) Cholangiogram through a right sided 14F biliary drain showing successful crossing of occluded biliary stent with opacification of biliary ducts (white arrow) as well as contrast passage into small bowel (black arrow). (D) Coronal slice from a contrast‐enhanced abdominal CT showing patent biliary stent (white arrow) with pneumobilia (black arrow) within intrahepatic biliary ducts indicating patency.
**Figure S2:** (A) Cholangiogram through previously placed biliary catheters in Patient 4 showing complete occlusion of the common hepatic duct (white arrow). Note ERCP scope within the small bowel/duodenum (black arrow). (B) Endoscopic image demonstrating the RF wire crossing the occlusion. (C) Cholangiogram through a right sided 14F biliary internal/external biliary drain catheter after successful crossing of occlusion showing opacification of intrahepatic biliary ducts (white arrow) as well as contrast opacification of small bowel (black arrow). (D) Over the wire cholangiogram showing opacification of intrahepatic biliary ducts (arrow) with passage of contrast into small bowel. There is no evidence of leak.

## Data Availability

The data that support the findings of this study are available from the corresponding author upon reasonable request.

## References

[1] M. G. House , J. L. Cameron , R. D. Schulick , et al., “Incidence and Outcome of Biliary Strictures After Pancreaticoduodenectomy,” Annals of Surgery 243, no. 5 (2006): 571–576, 10.1097/01.sla.0000216285.07069.fc. 16632990 PMC1570556

[2] K. M. Reid‐Lombardo , “Ramos‐De la Medina A, Thomsen K, Harmsen WS, Farnell MB. Long‐Term Anastomotic Complications After Pancreaticoduodenectomy for Benign Diseases,” Journal of Gastrointestinal Surgery 11, no. 12 (2007): 1704–1711, 10.1007/s11605-007-0369-7. 17929105

[3] C. Sperry , A. Malik , A. Reiland , et al., “Percutaneous Cystic Duct Interventions and Drain Internalization for Calculous Cholecystitis in Patients Ineligible for Surgery,” Journal of Vascular and Interventional Radiology 34, no. 4 (2023): 669–676, 10.1016/j.jvir.2022.12.468. 36581195

[4] C. Robins , N. Xiao , R. Salem , A. Malik , R. N. Keswani , and A. Riaz , “Percutaneous Biliary Neo‐Anastomosis or Neo‐Duct Creation Using Radiofrequency Wires,” Cardiovascular and Interventional Radiology 45, no. 3 (2022): 337–343, 10.1007/s00270-022-03059-5. 35106635

