# Supplementary material for: Successful Biliary Re‐Cannulation and Neo‐Anastomosis Creation in Complete Bile Duct Occlusion or Disruption Using a Combination of Interventional Radiology and Endoscopic Techniques: A Case Series
Source: JGH Open. 2026 Feb 24;10(2):e70278. doi: 10.1002/jgh3.70278 (PMC12932304; doi:10.1002/jgh3.70278)
Supplement: Supplementary file 1 — Figure S1: (A) Cholangiogram through a right sided external biliary drain in Patient 3 showing intrahepatic bile duct dilatation and complete occlusion of bile ducts (arrow). (B) Percutaneous access of occluded stent using 21G needle and 0.018 wire advanced through needle toward the biliary system (arrow). (C) Cholangiogram through a right sided 14F biliary drain showing successful crossing of occluded biliary stent with opacification of biliary ducts (white arrow) as well as contrast passage into small bowel (black arrow). (D) Coronal slice from a contrast‐enhanced abdominal CT showing patent biliary stent (white arrow) with pneumobilia (black arrow) within intrahepatic biliary ducts indicating patency. Figure S2: (A) Cholangiogram through previously placed biliary catheters in Patient 4 showing complete occlusion of the common hepatic duct (white arrow). Note ERCP scope within the small bowel/duodenum (black arrow). (B) Endoscopic image demonstrating the RF wire crossing the occlusion. (C) Cholangiogram through a right sided 14F biliary internal/external biliary drain catheter after successful crossing of occlusion showing opacification of intrahepatic biliary ducts (white arrow) as well as contrast opacification of small bowel (black arrow). (D) Over the wire cholangiogram showing opacification of intrahepatic biliary ducts (arrow) with passage of contrast into small bowel. There is no evidence of leak. [file JGH3-10-e70278-s001.docx]

**Figure legends**

**Figure 1.** A, Cholangiogram through a right sided external biliary drain in Patient 3 showing intrahepatic bile duct dilatation and complete occlusion of bile ducts (arrow). B, Percutaneous access of occluded stent using 21G needle and .018 wire advanced through needle towards the biliary system (arrow). C, Cholangiogram through a right sided 14F biliary drain showing successful crossing of occluded biliary stent with opacification of biliary ducts (white arrow) as well as contrast passage into small bowel (black arrow). D, Coronal slice from a contrast-enhanced abdominal CT showing patent biliary stent (white arrow) with pneumobilia (black arrow) within intrahepatic biliary ducts indicating patency.

**
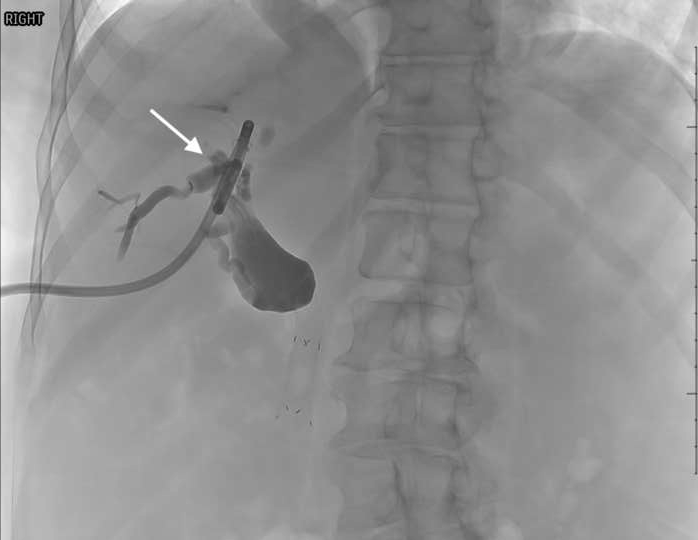
Fig. 1A**

**Fig. 1B**

**
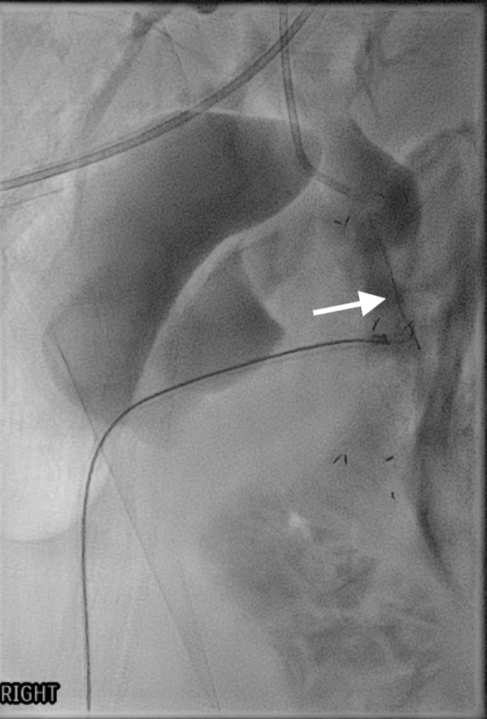
**

**
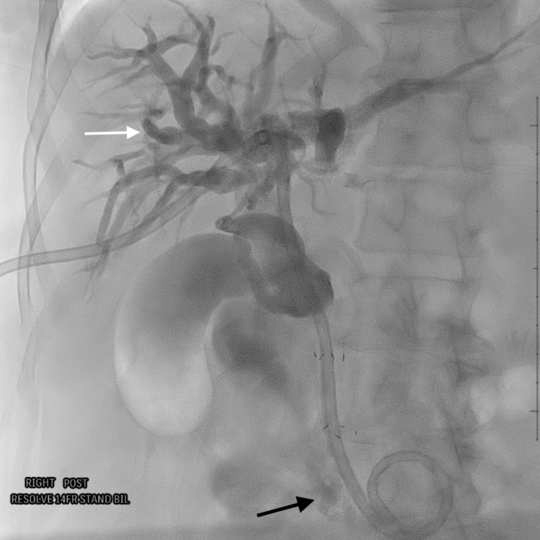
Fig. 1C**

**
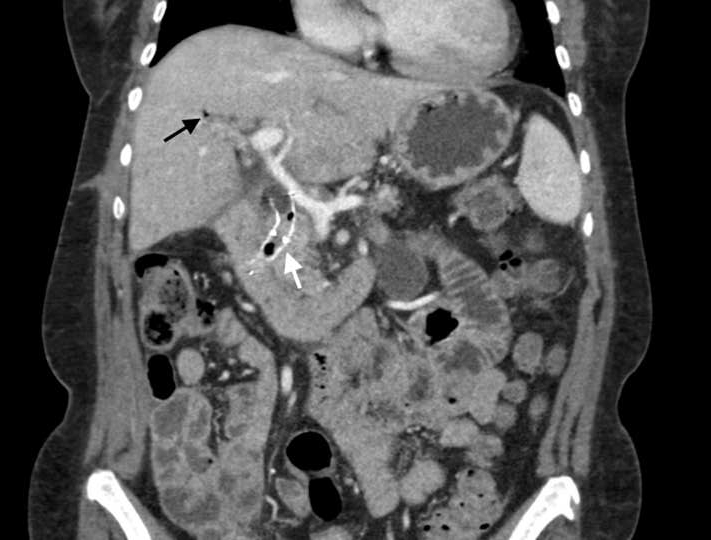
Fig. 1D**

***Supporting Information.***

**Supporting information legends**

**Figure 2.** A, Cholangiogram through previously placed biliary catheters in Patient 4 showing complete occlusion of the common hepatic duct (white arrow). Note ERCP scope within the small bowel/duodenum (black arrow). B, Endoscopic image demonstrating the RF wire crossing the occlusion. C, Cholangiogram through a right sided 14F biliary internal/external biliary drain catheter after successful crossing of occlusion showing opacification of intrahepatic biliary ducts (white arrow) as well as contrast opacification of small bowel (black arrow). D, Over the wire cholangiogram showing opacification of intrahepatic biliary ducts (arrow) with passage of contrast into small bowel. There is no evidence of leak.

**
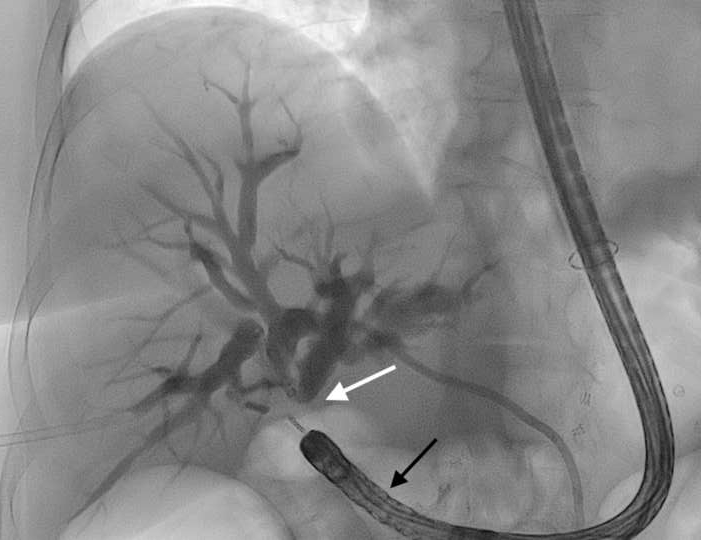
Fig. 2A**

**
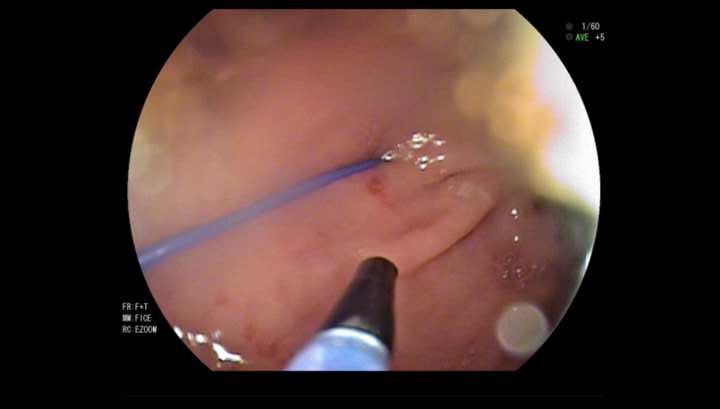
Fig. 2B**

**
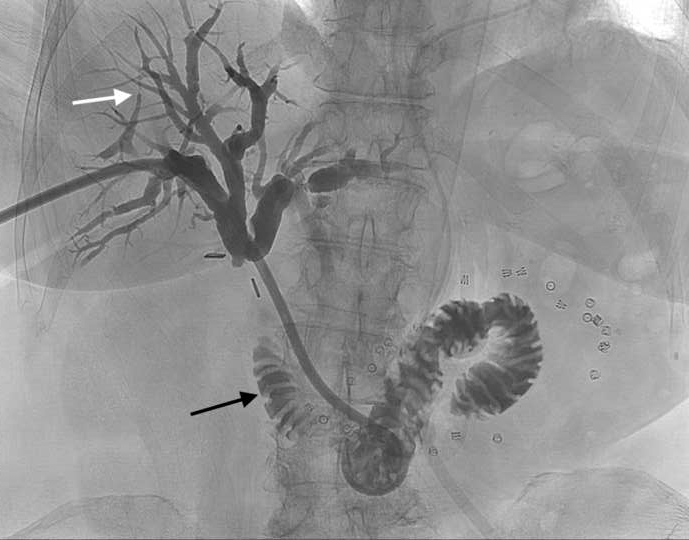
Fig. 2C**

**
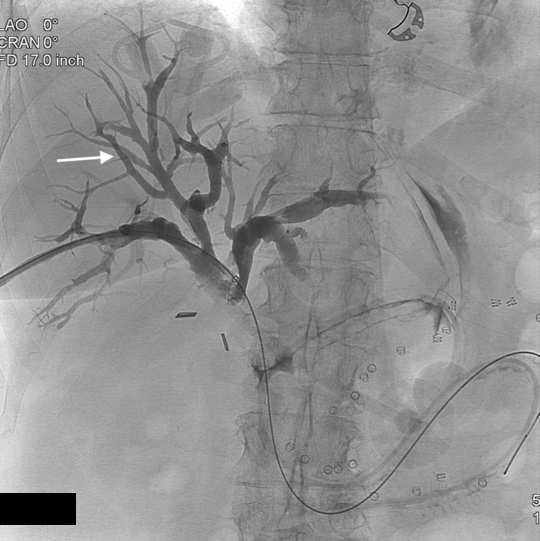
Fig. 2D**
